# Surbiton Cottage Hospital and the Sunday Fund Award

**Published:** 1916-08-26

**Authors:** R. Frederic Tyler

**Affiliations:** Acting Secretary, Surbiton Cottage Hospital. St. James's Road, Surbiton, Surrey


### THE EDITOR'S LETTER-BOX.

### Surbiton Cottage Hospital and the Sunday Fund Award.

*To the Editor of* The Hospital.

Sir,—I shall be obliged if you will make a correction in the next issue of The Hospital. In the list of amounts awarded to the various hospitals by the Metropolitan Hospital Sunday Fund we are put down as having received the sum of £35 9s. 2d., whereas the actual amount was 27 10s. 11d. Unless the correction is made it may cause questions to be asked when we publish our annual statement of accounts.

I am writing to the Secretary of the Fund, calling his attention to the discrepancy.

Thanking you in anticipation, I am, yours faithfully,
R. Frederic Tyler,
Acting Secretary, Surbiton Cottage Hospital.
St. James's Road,
Surbiton, Surrey, August 22, 1916.

[We are informed by the Secretary to the Metropolitan Hospital Sunday Fund that the sum awarded to the Surbiton Cottage Hospital by the Fund was, as printed in last week's issue, £35 9s. 2d. Only £27 10s. 11d. was paid over, as the balance of £7 18s. 3d. which was collected locally is paid to the hospital direct. —Ed. The Hospital.]

### Nurses' Indoor Uniform in the Streets: Unhygienic and Unprofessional.

*To the Editor of* The Hospital.

